# Symptom Burden in Postural Orthostatic Tachycardia Syndrome (POTS) and Endometriosis: A Retrospective Cohort Study

**DOI:** 10.7759/cureus.115147

**Published:** 2026-08-25

**Authors:** Adeline Y Chin, Ryan G Rilinger, Phoebe Leboit, Mackaleigh A Levine, Amy Nowacki, Ashley R Brant, Cheryl Cameron, Ashley Gubbels, Anna K Hayburn, Robert Wilson

**Affiliations:** 1 Neuromuscular Center, Department of Neurology, Cleveland Clinic, Cleveland, USA; 2 Neuromuscular Center, Department of Neurology, Cleveland Clinic Lerner College of Medicine, Cleveland, USA; 3 Department of Neurology, Cleveland Clinic Foundation, Cleveland, USA; 4 Department of Quantitative Health Sciences, Cleveland Clinic, Cleveland, USA; 5 Obstetrics and Gynecology Institute, Cleveland Clinic, Cleveland, USA; 6 Department of Nutrition, Case Western Reserve University School of Medicine, Cleveland, USA; 7 Section of Minimally Invasive Gynecologic Surgery and Chronic Pelvic Pain, Obstetrics and Gynecology Institute, Cleveland Clinic, Cleveland, USA; 8 Neuromuscular Center, Department of Neurology, Cleveland Clinic Foundation, Cleveland, USA

**Keywords:** anxiety, depression, endometriosis, postural orthostatic tachycardia syndrome (pots), symptom burden

## Abstract

Objective: The objective of this study is to evaluate differences in patient-reported symptom outcomes among individuals with postural orthostatic tachycardia syndrome (POTS) stratified by the presence or absence of co-occurring endometriosis.

Methods: We conducted a retrospective cohort analysis of 458 POTS patients evaluated at our autonomic center between 2018 and 2024. POTS diagnosis required documented orthostatic intolerance with a heart rate elevation of ≥30 beats per minute during the initial 10 minutes of head-up tilt table testing. Endometriosis was confirmed through surgical pathology reports where available, or via clinical documentation from a gynecology specialist. Patient-reported outcome measures were then compared between those with and without concurrent endometriosis.

Results: Among 1,322 female POTS patients, 229 (17.3%) also carried a diagnosis of endometriosis. Those with concurrent endometriosis demonstrated greater autonomic symptom severity, evidenced by elevated Composite Autonomic Symptom Score 31 (COMPASS-31) scores across several domains. Although the prevalence of anxiety and depression did not differ significantly between groups, both cohorts exhibited substantially higher rates than the general population.

Conclusion: Co-occurring endometriosis was associated with heightened autonomic dysfunction across several symptom domains, indicating that endometriosis may amplify the severity of POTS-related complaints. Both POTS groups demonstrated substantially elevated rates of anxiety and depression relative to the general population, revealing a considerable mental health burden irrespective of comorbid endometriosis. These results emphasize the need for thorough autonomic symptom evaluation and routine mental health screening in patients with POTS.

## Introduction

Postural orthostatic tachycardia syndrome (POTS) is an autonomic disorder estimated to affect between 300,000 and three million people across the United States [1]. The hallmark of POTS is characterized by an excessive heart rate increase upon standing (≥30 bpm within 10 minutes) in the absence of orthostatic hypotension and is accompanied by orthostatic intolerance, fatigue, cognitive difficulties, and gastrointestinal complaints [2,3]. Beyond these physical manifestations, patients with POTS frequently experience significant mental health challenges, including anxiety, depression, and reduced quality of life [4]. Sympathetic nervous system overdrive, neurotransmitter dysregulation, and central sensitization are potential biological contributors. These psychological symptoms are not only common but can exacerbate physical symptom burden, complicate daily functioning, and influence clinical management decisions, underscoring the need for comprehensive assessment of both autonomic and mental health domains in this population [5].

Endometriosis is a chronic gynecologic disorder defined by the growth of endometrial-like tissue in ectopic locations, commonly manifesting as pelvic pain, dysmenorrhea, dyspareunia, and reduced fertility [6-8]. Much like POTS, endometriosis exerts a substantial toll on both quality of life and psychological well-being. Affected individuals frequently experience anxiety, depression, fatigue, and limitations in social and occupational activities [9,10]. The convergence of persistent pain, hormonal dysregulation, and psychological distress underscores the multifaceted biopsychosocial impact of this condition [11].

Given the overlapping symptom profiles and the possibility that these conditions may compound one another’s effects on patient well-being, this retrospective cohort study sought to characterize differences in autonomic symptom severity and mental health between POTS patients with and without concurrent endometriosis. Outcomes were assessed using the COMPASS-31, PROMIS Fatigue and Global Health domains, PHQ-9, and GAD-7 instruments. Understanding these differences may guide the development of individualized treatment strategies and promote integrative care approaches that address both the physical and psychological dimensions of these conditions.

## Materials and methods

Patient identification and data collection

This retrospective cohort study was conducted at Cleveland Clinic (Cleveland, Ohio, USA), a tertiary academic medical center. The study cohort comprised all patients seen for an initial evaluation by a POTS specialist from January 1, 2018, through December 31, 2024. Institutional Review Board approval was obtained from the Cleveland Clinic (FWA00005367, Protocol #25-157). Informed consent was not required because the study involved only secondary use of existing clinical data and posed minimal risk to participants.

**Figure 1:**
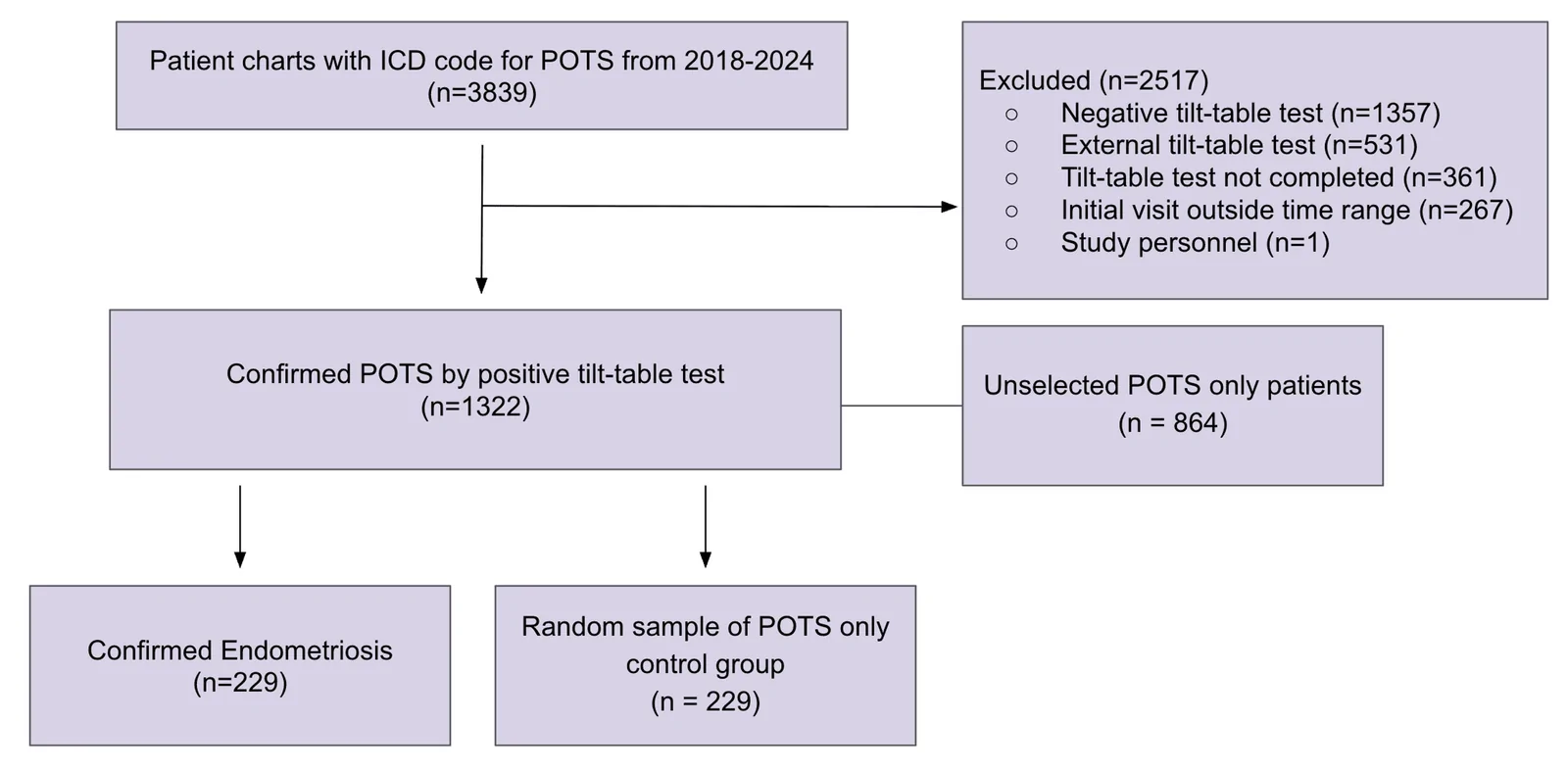
CONSORT flowchart diagram POTS: Postural orthostatic tachycardia syndrome

Epic SlicerDicer (Epic Systems Corporation, Verona, WI, USA) was used to identify 3,839 patient records with International Classification of Diseases (ICD) codes for POTS. Included patients were female, ≥18 years at the time of the head-up tilt table test (HUTT), and had a POTS diagnosis confirmed by a Cleveland Clinic specialist, defined as orthostatic intolerance symptoms with a heart rate elevation of ≥30 bpm within 10 minutes of HUTT in the absence of orthostatic hypotension. We excluded patients whose tilt table tests were negative, conducted at external facilities, or not completed. Of the 229 patients with co-occurring endometriosis, diagnostic confirmation was surgical in 146 cases (63.8%), imaging-based (ultrasound or MRI) in nine cases (3.9%), and clinical by a gynecology specialist in 74 cases (32.3%). A 1:1 age-matched control group of 229 female POTS-only patients was selected using a random number generator (Figure 1).

Patient-reported measures

During their initial evaluation with a POTS specialist, patients were prompted to complete validated questionnaires assessing autonomic symptom severity, fatigue, and psychological well-being.

Autonomic symptom burden was quantified using the Composite Autonomic Symptom Score 31 (COMPASS-31), a validated self-report questionnaire that evaluates six domains: orthostatic intolerance, vasomotor, secretomotor, gastrointestinal, bladder, and pupillomotor function. COMPASS-31 yields a total score ranging from 0 to 100, with higher values reflecting greater symptom severity [12].

The Patient-Reported Outcomes Measurement Information System (PROMIS) Fatigue T-score was used to evaluate fatigue severity, where elevated scores correspond to more pronounced fatigue. PROMIS measures have been validated in a range of clinical populations, including those with chronic conditions and autonomic dysfunction [13].

Anxiety and depression symptoms were assessed using two established instruments. The Generalized Anxiety Disorder-7 (GAD-7) scale, which generates scores from 0 to 21, was used to classify anxiety severity as minimal (<5), mild (5-9), moderate (10-14), or severe (15-21) [14]. Depression was assessed with the Patient Health Questionnaire-9 (PHQ-9), scored from 0 to 27, with severity classified as minimal (<5), mild (5-9), moderate (10-14), moderately severe (15-19), or severe (20-27) [15]. Patients with missing GAD-7 or PHQ-9 survey data were excluded from analysis.

Statistical analysis

Study data were captured and managed electronically through Research Electronic Data Capture (REDCap), a secure web-based platform for research data management [16]. Statistical analyses were performed in R version 4.4.1 [17] using RStudio version 2025.05.1+513.

Continuous variables, including patient demographics, COMPASS-31 domain and total scores, PROMIS fatigue T-scores, and PHQ-9 and GAD-7 scores, were compared between groups using Wilcoxon rank-sum tests. Categorical comparisons of race and the distributions of GAD-7 and PHQ-9 severity categories were performed using chi-squared tests. Two-sample proportion tests evaluated whether rates of anxiety and depression in our cohort differed from published rates among patients with chronic illnesses. Statistical significance was defined as p < 0.05.

## Results

We identified 1,322 female patients with tilt table-confirmed POTS. Of these, 229 (17.3%) had a concurrent endometriosis diagnosis. Baseline demographic characteristics, including age, race, and BMI, were comparable between groups (Table 1).

**Table 1:** Patient characteristics Age and BMI are reported as median (IQR), compared using the Wilcoxon rank-sum test. Race is reported as count (%), compared using the chi-squared test. POTS: Postural orthostatic tachycardia syndrome

	POTS only (n=229)	POTS and endometriosis (n=229)	Test statistic	p-value
Age (years)	30 (23-39)	31 (24-39)	W=26738	0.72
BMI (kg/m^2^)	23.8 (20.7-27.3)	24.6 (21.5-29.7)	W=28358	0.13
Race (% white)	203 (88.6%)	207 (90.4%)	χ²=0.209	0.65

**Figure 2:**
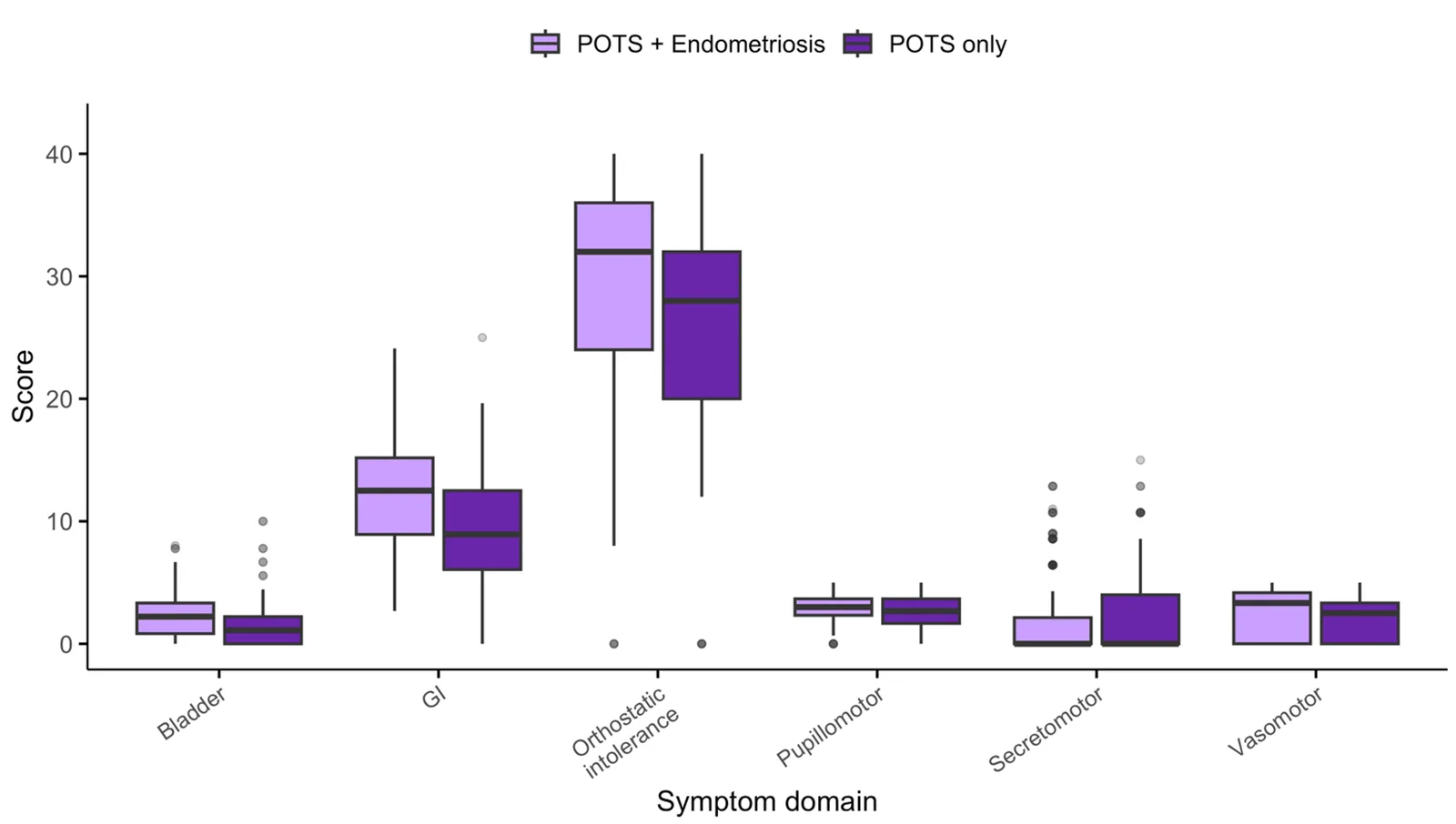
COMPASS-31 autonomic symptom domains in POTS patients with and without endometriosis POTS: Postural orthostatic tachycardia syndrome; COMPASS-31: Composite Autonomic Symptom Score 31

Patients with concurrent POTS and endometriosis exhibited greater autonomic symptom severity compared to those with POTS alone, as demonstrated by elevated COMPASS-31 scores in the orthostatic intolerance, vasomotor, gastrointestinal, bladder, and pupillomotor domains (Figure 2). The median total COMPASS-31 score was significantly higher in the endometriosis group (55.7 (47.7-64.8) vs. 48.8 (37.4-56.8); p = 2.89 × 10⁻⁶). Secretomotor domain scores were not significantly different between groups (Table 2).

**Table 2:** COMPASS-31, PROMIS, PHQ-9, and GAD-7 scores in patients with POTS with and without endometriosis Scores reported as median (Q1-Q3), compared using Wilcoxon rank-sum tests. POTS: Postural orthostatic tachycardia syndrome; COMPASS-31: Composite Autonomic Symptom Score 31; PROMIS: Patient-Reported Outcomes Measurement Information System; GAD-7: Generalized Anxiety Disorder-7

	POTS only (n=229)	POTS and Endometriosis (n=229)	Test statistic (W)	p-value
Orthostatic Intolerance	28 (20-32)	32 (24-36)	12459	0.02
Vasomotor	2.5 (0-3.33)	3.33 (0-4.17)	12674	0.007
Secretomotor	0 (0-4)	0 (0-2.14)	9031	0.80
GI	8.93 (6.06-12.5)	12.5 (8.93-15.2)	14245	<0.001
Bladder	1.11 (0-2.22)	2.22 (0.832-3.33)	12835	0.003
Pupillomotor	2.67 (1.67-3.67)	3 (2.33-3.67)	12424	0.02
COMPASS-31 score	48.8 (37.4-56.8)	55.7 (47.7-64.8)	14168	<0.001
PROMIS fatigue T-score	64 (61.2-69.5)	67 (57-76)	460	0.39
PHQ-9 score	9 (5-13)	10 (5.5-13)	21540	0.37
GAD-7 score	7 (4-13)	8 (3-13)	17708	0.50

**Figure 3:**
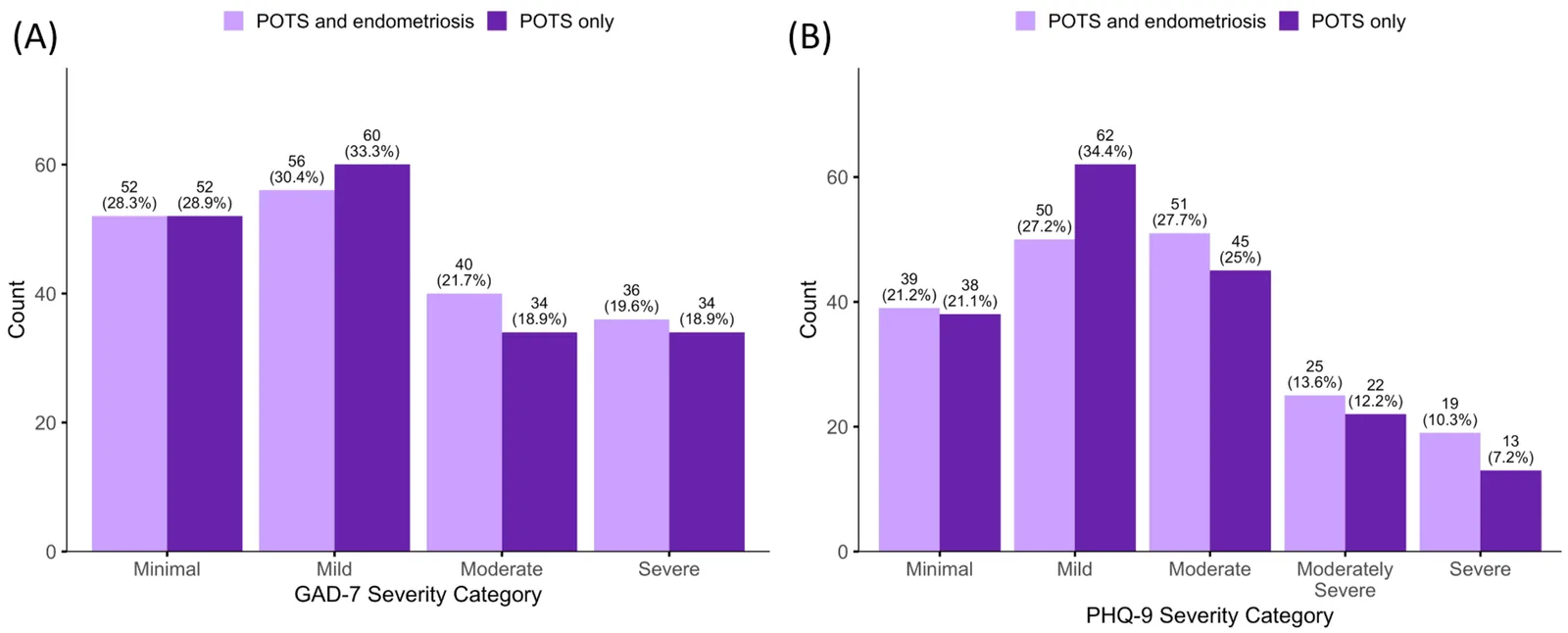
GAD-7 and PHQ-9 severity in POTS patients with and without endometriosis A: GAD-7 severity in POTS patients with and without endometriosis. Anxiety severity was classified as minimal (<5), mild (5-9), moderate (10-14), or severe (15-21). B: PHQ-9 severity in POTS patients with and without endometriosis. Depression severity was classified as minimal (<5), mild (5-9), moderate (10-14), moderately severe (15-19), or severe (20-27). POTS: Postural orthostatic tachycardia syndrome; PROMIS: Patient-Reported Outcomes Measurement Information System; GAD-7: Generalized Anxiety Disorder-7

PROMIS fatigue T-scores, PHQ-9 depression scores, and GAD-7 anxiety scores did not differ significantly between groups (Table 2). Similarly, the distribution of anxiety and depression severity categories was comparable between cohorts (p = 0.16 and p = 0.073, respectively; Figure 3). GAD-7 and PHQ-9 data from the initial visit to a Cleveland Clinic specialist were available for 184 of 229 POTS and endometriosis patients and 180 of 229 POTS-only patients.

We further evaluated anxiety and depression rates in our cohorts relative to published rates among chronic pain patients [18]. Anxiety was present in 55.9% of POTS patients with endometriosis and 48.9% of POTS-only patients, both significantly exceeding the 40.2% prevalence reported in chronic pain populations (p < 0.001). Depression affected 48.0% of the POTS and endometriosis group and 39.3% of the POTS-only group. Compared to chronic pain patients, depression rates were significantly elevated in the POTS and endometriosis group (p = 0.007) but not in the POTS-only group (p = 0.99).

## Discussion

This study represents one of the first retrospective cohort analyses examining autonomic symptom burden and mental health in POTS patients with or without concurrent endometriosis. Our findings demonstrate that individuals with co-occurring POTS and endometriosis experience more pronounced autonomic dysfunction spanning multiple COMPASS-31 domains, including orthostatic intolerance, vasomotor, gastrointestinal, bladder, and pupillomotor function. These results indicate that concurrent endometriosis may be linked to a heightened severity of POTS-related autonomic symptoms.

Notably, PROMIS fatigue, PHQ-9, and GAD-7 scores were not significantly different between groups, indicating that while autonomic symptom severity is amplified, the overall burden of fatigue, depression, and anxiety may not be further exacerbated by co-occurring endometriosis within this cohort. Nevertheless, the prevalence of anxiety and depression among POTS patients with endometriosis was substantially elevated compared to chronic pain populations [18], underscoring a meaningful mental health burden that endometriosis may compound. The physical manifestations of POTS, such as tachycardia, chest discomfort, dyspnea, dizziness, lightheadedness, tremor, and diaphoresis, can closely resemble anxiety symptoms, potentially fostering emotional distress; conversely, psychological stress may activate the autonomic stress response and exacerbate somatic symptoms [19]. Additional contributors to heightened anxiety in this population include central sensitization [20], the unpredictable nature of symptom flares [21], and a perceived lack of control over disease management [22].

Our observations are consistent with existing literature documenting elevated rates of autonomic and psychiatric comorbidity in POTS and point to a possible cumulative effect of endometriosis on autonomic symptom severity. Although the pathophysiologic basis of this association requires further investigation, potential mechanisms include chronic systemic inflammation, central pain sensitization, and the hormonal perturbations characteristic of endometriosis [11,23-25]. Individuals managing both conditions may face additional psychosocial stressors that adversely affect mental health, including experiences of medical invalidation [26], diminished occupational capacity [27], and strain on interpersonal relationships [28,29]. Such challenges can engender frustration, helplessness, social isolation, and a diminished sense of identity and self-worth.

Limitations

The retrospective design of this study limits our ability to establish a causal relationship between endometriosis and increased autonomic symptom severity. Prospective studies are necessary to determine whether endometriosis directly exacerbates autonomic dysfunction or whether these conditions share common pathophysiologic underpinnings.

As a single-center study conducted at a tertiary referral institution, our results may be influenced by referral bias and could represent a more medically complex patient population. Furthermore, our stringent tilt table-based diagnostic criteria for POTS resulted in only 34.4% of patients with a POTS-related ICD code meeting inclusion criteria, reflecting considerable variability in how providers define and document POTS diagnoses. This suggests that many patients carry a clinical POTS diagnosis without formal tilt table confirmation, and consequently, our findings may not be generalizable to patients diagnosed through alternative methods.

The reliance on patient-reported outcome measures obtained at a single time point introduces the potential for recall bias and individual variability in symptom reporting. While the instruments employed are well-validated and broadly utilized in clinical studies, they may not comprehensively capture the dynamic nature of autonomic dysfunction and mental health in this population. Longitudinal research is warranted to assess whether the between-group differences identified in our analysis are sustained over time.

## Conclusions

In this retrospective analysis, POTS patients with concurrent endometriosis had a significantly greater autonomic symptom burden, as evidenced by elevated total COMPASS-31 scores and higher domain scores for orthostatic intolerance, vasomotor, gastrointestinal, bladder, and pupillomotor function. No significant between-group differences were observed in PROMIS fatigue, PHQ-9, or GAD-7 scores, suggesting that the excess symptom burden associated with endometriosis may be predominantly autonomic in nature rather than attributable to fatigue, depression, or anxiety. However, anxiety and depression prevalence across both POTS groups surpassed rates documented in chronic pain populations, with the endometriosis group demonstrating significantly higher depression rates compared to chronic pain patients. These findings underscore the importance of thorough autonomic symptom evaluation in POTS patients with endometriosis and highlight the considerable mental health burden faced by this population. Future prospective studies are needed to delineate the pathophysiologic mechanisms driving the relationship between endometriosis and amplified autonomic dysfunction in POTS.
